# Supplementary material for: Fibroblast Growth Factor-23 (FGF-23) in Dogs—Reference Interval and Correlation with Hematological and Biochemical Parameters
Source: Animals (Basel). 2023 Oct 13;13(20):3202. doi: 10.3390/ani13203202 (PMC10603748; doi:10.3390/ani13203202)
Supplement: Supplementary file 1 [file animals-13-03202-s001.zip › animals-2570783-supplementary.pdf]

animals

Supplementary Table S1. Assessment of normal distribution for hematological and biochemical parameters as well as the fibroblast growth factor (FGF)-23 by Shapiro-Wilk-test in 40 dogs divided in groups I-IV based on creatinine concentration according to the guidelines of the International Renal Interest Society (IRIS).

Parameter	Study Group I	Study Group II	Study Group III	Study Group IV
Fibroblast growth factor-23 (N=40)[1]	0.391 (n=10)	0.480 (n=10)	0.593 (n=10)	<0.001 (n=10)
		Hematology[2]		
Red blood cell count (N=36)	0.152 (n=10)	0.531 (n=9)	0.497 (n=7)	0.283 (n=10)
Hemoglobin (N=36)	0.104 (n=10)	0.333 (n=9)	0.518 (n=7)	0.434 (n=10)
Hematocrit (N=36)	0.023 (n=10)	0.822 (n=9)	0.853 (n=7)	0.537 (n=10)
Reticulocytes (N=36)	0.065 (n=10)	0.452 (n=9)	0.324 (n=7)	0.026 (n=10)
Reticulocyte hemoglobin (N=36)	0.200 (N=10)	0.268 (n=9)	0.354 (n=7)	0.509 (n=10)
White blood cell count (N=36)	0.567 (n=10)	0.327 (n=9)	0.387 (n=7)	<0.001 (n=10)
Neutrophils (N=36)	0.068 (n=10)	0.210 (n=9)	0.429 (n=7)	0.823 (n=10)
Eosinophils (N=36)	0.008 (n=10)	0.032 (n=9)	0.652 (n=7)	0.022 (n=10)
Lymphocytes (N=36)	0.280 (n=10)	0.088 (n=9)	0.498 (n=7)	0.609 (n=10)
Monocytes (N=36)	0.782 (n=10)	0.046 (n=9)	0.397 (n=7)	0.091 (n=10)
Basophils (N=36)	<0.001 (n=10)	- (n=9)	- (n=7)	- (n=10)
Thrombocytes (N=36)	0.537 (n=10)	0.488 (n=9)	0.396 (n=7)	0.096 (n=10)
		Biochemistry[3]		
Creatinine (N=40)	0.105 (n=10)	<0.001 (n=10)	0.234 (n=10)	0.256 (n=10)
Urea (N=40)	0.815 (n=10)	0.004 (n=10)	0.454 (n=10)	0.429 (n=10)
Symmetric dimethylarginine (N=3)	- (n=0)	- (n=2)	- (n=0)	- (n=1)
Total calcium (N=40)	0.149 (n=10)	0.717 (n=10)	0.531 (n=10)	0.313 (n=10)
Phosphorus (N=40)	0.570 (n=10)	0.010 (n=10)	0.037 (n=10)	0.174 (n=10)
Potassium (N=40)	0.338 (n=10)	0.841 (n=10)	0.856 (n=10)	0.389 (n=10)
Sodium (N=40)	0.618 (n=10)	0.041 (n=10)	0.082 (n=10)	0.047 (n=10)
Magnesium (N=37)	0.002 (n=10)	0.041 (n=8)	0.222 (n=10)	0.405 (n=9)
Total protein (N=40)	0.637 (n=10)	0.257 (n=10)	0.170 (n=10)	0.002 (n=10)
Albumin (N=40)	0.058 (n=10)	0.755 (n=10)	0.333 (n=10)	0.482 (n=10)
Globulin (N=40)	0.006 (n=10)	0.433 (n=10)	0.969 (n=10)	0.005 (n=10)
Glucose (N=39)	0.673 (n=10)	<0.001 (n=9)	<0.001 (n=10)	0.492 (n=10)
Fructosamine (N=40)	0.381 (n=10)	0.025 (n=10)	0.025 (n=10)	0.258 (n=10)
Alpha-amylase (N=37)	0.144 (n=10)	0.705 (n=8)	0.324 (n=10)	<0.001 (n=9)
DGGR-lipase (N=40)	0.462 (n=10)	0.271 (n=10)	0.968 (n=10)	<0.001 (n=10)
Bilirubin (N=39)	0.668 (n=10)	0.763 (n=10)	0.088 (n=9)	0.263 (n=10)
Alanine aminotransferase (N=40)	0.362 (n=10)	0.042 (n=10)	<0.001 (n=10)	0.489 (n=10)
Alkaline phosphatase (N=40)	0.023 (n=10)	<0.001 (n=10)	<0.001 (n=10)	0.180 (n=10)
Aspartate aminotransferase (N=40)	0.392 (n=10)	<0.001 (n=10)	0.055 (n=10)	0.258 (n=10)

Glutamate dehydrogenase (N=40)	0.025 (n=10)	<0.001 (n=10)	<0.001 (n=10)	0.283 (n=10)
Creatine kinase (N=40)	<0.001 (n=10)	0.035 (n=10)	0.178 (n=10)	0.008 (n=10)
Triglycerides (N=37)	0.156 (n=10)	<0.001 (n=8)	0.168 (n=10)	0.068 (n=9)

Normal distribution was assessed if $P < 0.05$.

[1]FGF-23 ELISA Kit, Kainos Laboratories, Tokyo, Japan; [2]Sysmex XT 2000iv, Sysmex Deutschland, Germany; [3]COBAS 8000, Roche Diagnostics, Germany.
